# Transcriptomics, Proteomics and Network Pharmacology Reveal the Mechanisms of Cantharidin-Induced Kidney Injury in Rats

**DOI:** 10.3390/cimb48050460

**Published:** 2026-04-29

**Authors:** Xiaotong Duan, Cancan Zhao, Sali Li, Liu Liu, Ming Yu, Yuanming Wu, Jianyong Zhang, Xiaofei Li

**Affiliations:** 1 School of Basic Medicine, Guizhou Medical University, No. 6 Ankang Avenue, Guiyang 561113, China; dxt199747@163.com (X.D.);; 2 School of Basic Medicine, Zunyi Medical University, No. 6 Xuefu West Road, Zunyi 563000, China; 3 Key Laboratory of Basic Pharmacology Ministry Education, Zunyi Medical University, No. 6 Xuefu West Road, Zunyi 563000, China; 4 Joint International Research Laboratory of Ethnomedicine Ministry of Education, Zunyi Medical University, No. 6 Xuefu West Road, Zunyi 563000, China; 5 State Key Laboratory of Functions and Applications of Medicinal Plants, Guizhou Natural Products Research Center, Guiyang 550014, China

**Keywords:** cantharidin, kidney injury, transcriptomics, proteomics, network pharmacology, mechanism

## Abstract

Cantharidin (CTD), the principal active constituent of the traditional Chinese medicine (TCM) Mylabris, exhibits potent antitumor activity. However, its clinical application is limited by organ toxicity (especially nephrotoxicity), and the underlying mechanisms remain incompletely defined. This research endeavored to elucidate the nephrotoxic effects and molecular mechanisms of CTD in rats using serum biochemical assays, histopathological examinations, and a multi-omics strategy. CTD treatment significantly increased levels of urea nitrogen and creatinine and induced histopathological injury in the kidneys. Transcriptomics, proteomics, and network pharmacology integrated analysis have revealed 14 common targets, mainly involved in the glutathione metabolic pathway. Further verification showed that CTD markedly upregulated protein expression of glutaminase (GLS), while downregulating homogentisate 1,2-dioxygenase (HGD), glutamate-cysteine ligase catalytic subunit (GCLC), and regulatory subunit (GCLM), thereby suppressing the glutathione metabolic pathway and exacerbating kidney injury. Our results indicate that CTD induces oxidative stress and consequent nephrotoxicity by inhibiting the glutathione metabolic pathway, providing a mechanistic basis for optimizing clinical strategies to mitigate CTD-induced kidney injury.

## 1. Introduction

Cantharidin (CTD) is an important active component extracted from *Mylabris* [1] and exerts notable anti-tumor effects by inducing cell cycle arrest and apoptosis while inhibiting autophagy [2,3]. Multiple CTD-based pharmaceutical formulations, including disodium cantharidinate injection and compound *Mylabris* capsules, have demonstrated effective anti-tumor activity against lung cancer [4], gastric cancer [5], pancreatic cancer [6,7], and liver cancer [8]. However, the therapeutic dose of CTD is very close to its toxic dose, with an oral lethal dose ranging from 10 to 60 mg and a median lethal dose (LD_50_) of 1.71 mg/kg in mice [9]. Accumulating evidence indicates that CTD induces hepatotoxicity [10], nephrotoxicity [11], and urinary system damage [12]. Further studies have found that kidney injury accounts for 26.40% of total CTD toxicity cases, presenting clinically as nephritis, acute renal failure, acute renal insufficiency, toxic renal damage, and acute kidney injury [13].

Studies have identified several mechanisms underlying CTD-induced kidney injury, including apoptosis [14], inhibition of protein phosphatase 2A [15], endoplasmic reticulum stress, autophagy activation [16], oxidative injury, and inflammation [17]. Additionally, CTD disrupts levels of phosphatidylethanolamine, phosphatidylcholine, lysophosphatidylcholine, and sphingomyelin, ultimately inhibiting glycerophospholipid and sphingolipid metabolic pathways in kidney injury [18]. Nevertheless, the key molecular targets and regulatory networks underlying CTD-induced kidney injury remain incompletely understood.

Transcriptomics and proteomics are powerful tools for uncovering the underlying and surface factors of biological phenomena, respectively [19]. Integration of transcriptomics and proteomics has been widely applied in pharmacological and toxicological research, including target identification, drug resistance, biomarker screening, and signaling pathways [20,21]. Notably, recent studies have combined transcriptomics, proteomics, and network pharmacology to reveal that Yiqi Tongluo Granule protects against cerebral ischemia/reperfusion injury by inhibiting neuronal apoptosis enhanced by the PI3K-Akt signaling pathway [22,23]. Therefore, combining transcriptomics, proteomics, and network pharmacology allows for an in-depth gene/protein-level analysis of biological processes, followed by molecular biology verification, thereby comprehensively elucidating the mechanisms of CTD-induced kidney injury.

In this study, histopathological observation and biochemical detection were preliminarily used to evaluate the damaging effects of CTD on the kidney tissue of rats. Subsequently, transcriptomics, proteomics, network pharmacology, and molecular docking were employed to screen potential targets. Finally, the effects of CTD on the key targets were verified using molecular docking and Western blotting (WB) techniques. In conclusion, our results will provide new scientific evidence for the prevention and treatment of CTD-induced kidney injury.

## 2. Materials and Methods

### 2.1. Animal Experiment Design and Drug Administration

Specific pathogen-free (SPF) male Sprague Dawley rats (weighing 200 ± 10 g) were purchased from Chongqing Tengxin Biotechnology Co., Ltd., Chongqing, China (License No.: SCXK (Beijing) 2019-0010). The study protocol was approved by the Animal Ethics Committee of Zunyi Medical University ((2021) 2-139), and carried out in accordance with international guidelines for the care and use of laboratory animals.

CTD (CAS:56-25-7, purity > 98%) was purchased from Beijing SL Pharmaceutical Co., Ltd. (Beijing, China). Firstly, 15 mg of CTD was dissolved in 100 mL of 0.5% CMC-Na solution for 10 min under 100 MHz sonication. After mixing, a 0.15 mg/mL CTD suspension was prepared and stored at 4 °C.

The rats were adaptively fed for 7 days in a specific pathogen-free room at 22 °C ± 2 °C and 40–60% relative humidity, under a 12 h light/dark cycle. Twenty male SD rats were randomized into the control group and the CTD (1.5 mg·kg^−1^) groups (10 rats each group). The drugs (CTD suspended in a 0.5% carboxymethylcellulose) were administered by gavage daily for 28 days. The control rats received an equivalent volume of 0.5% carboxymethylcellulose. All rats had ad libitum access to sterile food and water. At the end of the experiment, blood samples and kidney tissue were prepared for follow-up studies. The kidney tissue samples were fixed in 4% paraformaldehyde or stored at −80 °C for further analysis.

### 2.2. Serum Biochemical Indexes Detection

The blood samples were spun at 1000 *g* (Sorvall ST 16R, Thermo Fisher Scientific, Bohemia, NY, USA) for 15 min at 4 °C to obtain the serum. The levels of serum creatinine (Scr) and blood urea nitrogen (BUN) were examined by an automatic biochemical analyzer (AU680, Beckman Coulter, Inc., Brea, CA, USA).

### 2.3. Histopathological Examination (H&E)

Rat kidney tissues were fixed in 4% paraformaldehyde for 24 h and processed with routine histology procedures. Subsequently, kidney tissue sections were stained with haematoxylin and eosin (H and E) according to standard protocol for histopathological assessment using a microscope (Nikon, Tokyo, Japan).

### 2.4. Transcriptomics Assay

Total RNA from kidney samples were extracted by Trizol (AJ91799A, TakaRa, Kusatsu, Japan) in accordance with the specification, and the RNA purity was inspected by the RNA 1000 Nano LabChip Kit (Agilent, Santa Clara, CA, USA). Establishment of the databases and RNA-seq were executed by APTBIO Technology Co., Ltd., Shanghai, China (https://bio-cloud.aptbiotech.com/plus/#/login (accessed on 14 November 2022)). In short, mRNAs were broken into pieces, and then the RNA fragments were reverse transcribed to produce the ending cDNA library following the mRNASeq sample preparation kit (Illumina, San Diego, CA, USA). Ultimately, sequencing was performed with the Illumina Hiseq 4000 (LC Sciences, Houston, TX, USA) platform. StringTie was used to perform the expression level for mRNAs by calculating fragments per kilobase of transcript per million fragments mapped (FPKM). The differentially expressed genes (DEGs) were selected with log2 |fold change| ≥ 1, with a statistical significance (*p*-value ≤ 0.05) by R package. The Kyoto Encyclopedia of Genes and Genomes (KEGG) and Gene Ontology (GO) databases were used to classify and group these DEGs.

### 2.5. Proteomics Assay

An automated tissue homogenizer (HG-400 MiniG, SPEX Sample Prep, Metuchen, NJ, USA) was used to extract total protein with protein lysis solution (Cat. No. P0013G, Beyotime, Shanghai, China) from the rat kidney. The Q Exactive Orbitrap Mass Spectrometer equipped with UltiMate 3000 RSLCnano System (C18 column, 3 μm, 0.075 × 150 mm, Thermo Fisher, USA) was employed to capture data in this research. The isolation procedure of peptides was as follows: 0 min, 98% A (0.1% formic acid in water), 2% B (0.1% formic acid in acetonitrile); 5 min, 95% A, 5% B; 50 min, 65% A, 35% B; 55 min, 2% A, 98% B; 60 min, 98% A, 2% B. The peptides were detected in the positive ion mode of the mass spectrometer. The tests were executed by APTBIO Technology Co., Ltd., Shanghai, China.

MaxQuant (Version 1.5.3.17) was used for the identification and quantitative analysis of mass spectrometry raw data, and the parameters were set as follows: for precursor ion scans, the mass tolerance was set to 6 ppm, and for production scans, the mass tolerance was 20 ppm. Cysteine carbamidomethylation was designated as a fixed modification, and oxidation of methionine was included as a variable modification. The false discovery rate (FDR) for peptides was maintained at ≤1%. A maximum of two miscleavage sites was allowed. The MaxQuant software (Version 1.5.3.17) normalized the data using the total sum intensity strategy. The ComplexHeatmap package in R language (Version 3.4) was used to classify the expression of proteins, and differentially expressed proteins (DEPs) were screened by FC > 2 or FC < 0.5 with *p* < 0.05. The Kyoto Encyclopedia of KEGG and GO analysis were completed using the DAVID database to explore the biological functions and potential enrichment pathways.

### 2.6. Network Pharmacology Analysis

The TCMSP database (https://tcmsp-e.com/tcmspsearch.php (accessed on 12 March 2025)) was used to collect targets of active ingredients in CTD. Targets associated with “renal injury”, “kidney injury”, “renal toxicity” and “nephrotoxicity” disease names were collected through GeneCards (https://www.genecards.org/ (accessed on 12 March 2025)), the Digsee database (http://210.107.182.61/geneSearch/ (accessed on 12 March 2025)), and the Comparative Toxicogenomics Database (http://ctdbase.org/ (accessed on 12 March 2025)). Then, the total of ingredient targets was obtained by combining and removing duplicates from the three databases. Finally, the Venn software mapping tool platform (https://bioinfogp.cnb.csic.es/tools.html (accessed on 12 March 2025)) was used for collecting common targets, and targets at the intersection of CTD ingredient targets and kidney injury-related targets were obtained as potential therapeutic targets. These overlapping targets were imported into UniProt (https://www.uniprot.org/ (accessed on 12 March 2025)) to standardize the gene names. All the network-predicted targets of CTD-induced kidney injury were introduced into the STRING (http://string-db.org (accessed on 13 March 2025)) database to conduct a PPI analysis. The species was limited to Homo sapiens, filtered through the minimum required interaction score, and the highest confidence (0.9) set as the threshold. Then, Cytoscape 3.9.0 software and its plug-in CytoNCA were used to conduct further analysis and embellishment on the PPI network.

### 2.7. Bioinformatics Integration Analysis

The intersection of DEGs, DEPs, and network pharmacology-derived targets was taken to identify significant targets of CTD-induced kidney injury. Hierarchical clustering was used to group and classify the significant targets (data standardization method: z-score). GO functional enrichment and KEGG pathway analysis of significant targets were performed using the DAVID database to obtain relevant biological functions and disturbed metabolic pathway.

### 2.8. Molecular Docking

The targets obtained from PPI analyses of significant targets were integrated to screen core targets for CTD-induced kidney injury. The atomic coordinates of the pivotal target were obtained from the PDB (http://www.rcsb.org/ (accessed on 14 March 2025)) and processed in AutoDockTools 1.5.6. The original position of the protein ligand was selected as the candidate ligand space. Then, the original ligand was extracted to generate the active pocket, and the search grid expansion was set to 6 Å for docking. The proteins were then subjected to additional treatments, such as the addition of hydrogen atoms, removal of water molecules, and energy optimization. The 3D structure of CTD, sourced from the PubChem Database (https://pubchem.ncbi.nlm.nih.gov/ (accessed on 14 March 2025)), was constructed in AutoDockTools 1.5.6, incorporating calculations of atomic partial charges and parameterization. Subsequently, PyMOL 2.7.2 was used to visualize the interacting amino acids, and the length and number of hydrogen bonds.

### 2.9. Western Blotting

Approximately 50 mg of rat kidney tissue was collected, 500 μL of lysis buffer was added, and then the mixture was homogenized in a grinder. The tissue homogenate was allowed to incubate on ice for 20–30 s, followed by centrifugation at 19,500× *g* for 10 min at 4 °C. The supernatant was transferred into an Eppendorf^®^ tube. The total protein content was evaluated using a BCA assay, electrophoretically separated using a 7.5% gel and subsequently transferred to a polyvinylidene difluoride (PVDF) membrane. For blocking, 5% skimmed milk was used. The membrane was then incubated overnight at 4 °C with polyclonal primary antibodies. The antibodies against GCLC (1: 2000, Cat No. 12601-1-AP), HGD (1: 5000, Cat No. 16465-1-AP), GCLM (1: 2000, Cat No. 14241-1-AP), GLS (1: 5000, Cat No. 12855-1-AP) and GAPDH (1: 10000, Cat No. 10494-1-AP) were purchased from Proteintech Group (Wuhan, China). The PVDF membrane was rinsed four times (10 min each) with TBST (1×), followed by a 2 h incubation with the appropriate secondary antibody solution. Finally, the membrane was immersed in an ECL developer, and detection was carried out using the BIO-RAD Chemiluminescent Imager and Image Lab software (Version 3.0).

### 2.10. Statistical Analysis

Experimental results were presented as mean ± standard deviation. Data were statistically analyzed using the Statistical Package for the Social Sciences software (version 29.0). Differences between two groups were analyzed by independent sample t-test. Levene’s test was used to assess variance homogeneity, and the Games–Howell test was applied when variances were unequal. *p* < 0.05 and *p* < 0.01 were considered statistically significant.

## 3. Results

### 3.1. CTD-Induced Kidney Injury in Rats

**Figure 1:**
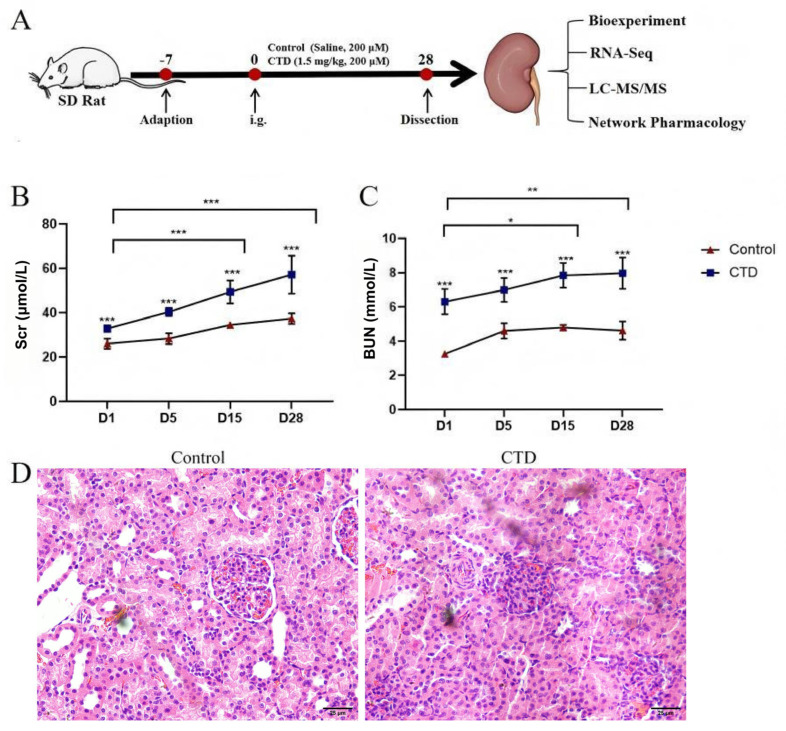
CTD-induced kidney injury in rats. (**A**) The general experimental procedure of CTD-induced kidney injury in rats. After 28 d of CTD stimulation, kidneys were collected for subsequent biological experiments. The levels of Scr (**B**) and BUN (**C**) in the rat serum after CTD stimulation ($\bar{x}$ ± SD, *n* = 3, * represents *p* < 0.05, ** represents *p* < 0.01, *** represents *p* < 0.001). (**D**) Representative images of H and E staining in the kidney (×400).

After rats were exposed to CTD (Figure 1A), serum levels of Scr and BUN were significantly increased compared with the control group (*p* < 0.05) after 5 days of CTD stimulation (Figure 1B,C). Histopathological examination revealed moderate focal renal tubular injury in the CTD group (Figure 1D), characterized by marked inflammatory infiltration in the renal interstitium, focal necrosis of tubular epithelial cells, diffuse vacuolar degeneration, and loss of brush borders in proximal tubules. The renal corpuscles remained largely intact with only mild congestion, confirming that CTD-induced kidney injury is predominantly tubular in nature.

### 3.2. Transcriptomic Analysis of CTD-Induced Kidney Injury

**Figure 2:**
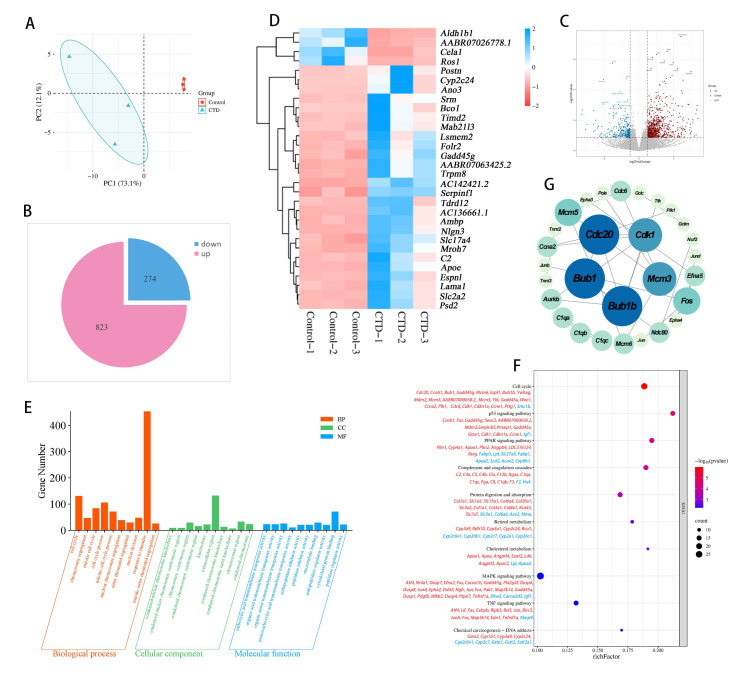
Transcriptomic analysis of CTD-induced kidney injury. (**A**) Principal component analysis. (**B**) Pie plot showing the number of differentially expressed genes (CTD vs. control). (**C**) Volcano plot of differential expressed genes. (**D**) Heat map of DEGs (blue: downregulated genes, red: upregulated genes). (**E**) Three functional categories in the GO analysis. (**F**) KEGG pathway analysis of DEGs between the CTD group and control group (blue: downregulated genes, red: upregulated genes). (**G**) PPI analysis of DEGs.

RNA sequencing was performed on kidney tissues collected at 28 days. Principal component analysis (PCA) was applied to assess the reproducibility of gene expression profiles (Figure 2A). We found 1097 DEGs in the kidneys of the CTD group, of which 823 DEGs were upregulated and 274 DEGs were downregulated (Figure 2B,C). Cluster analysis is shown in Figure 2D. GO analysis of the DEGs indicated that the top 10 biological processes included cell cycle, chromosome segregation, and mitotic cell cycle (Figure 2E). KEGG analysis enriched the top 10 signaling pathways including cell cycle, p53, PPAR, MAPK, and TNF signaling pathways (Figure 2F).

The PPI network was constructed by inputting 1094 DEGs into the STRING database, selecting the rattus norvegicus species, setting the minimum interaction score to high confidence (0.900), and removing disconnected nodes in the network. Topological parameters were analyzed using the CytoNCA2.1.6, and core target proteins were screened based on betweenness centrality (BC > 484.96), closeness centrality (CC > 0.0056), and degree centrality (DC > 25.5). The results showed that five genes, namely *bub1b*, *bub1*, *cdc20*, *mcm3* and *cdk1*, ranked the highest and may be the key targets involved in CTD-induced kidney injury (Figure 2G).

### 3.3. Proteomic Analysis of CTD-Induced Kidney Injury

**Figure 3:**
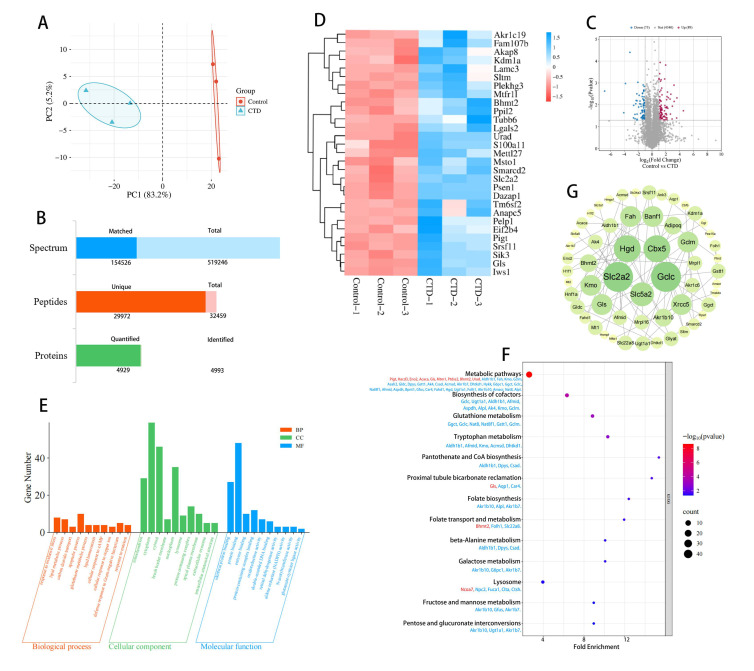
Proteomic analysis of CTD-induced kidney injury. (**A**) Principal component analysis. (**B**) Statistical chart of identification and quantitative results. (**C**) Volcano plot of differentially expressed proteins. (**D**) Heat map of DEPs. (**E**) Three functional categories in the GO analysis. (**F**) KEGG pathway analysis of DEPs between the CTD group and control group (blue: downregulated genes, red: upregulated genes). (**G**) PPI analysis of DEPs.

PCA was conducted to assess the reproducibility of gene expression profiles and the separation between experimental groups (Figure 3A). A total of 519,246 secondary spectra were acquired, yielding 154,526 matched spectra, 32,459 peptides, 4993 identified proteins, and 4929 quantified proteins (Figure 3A). Volcano plots were employed to compare differential proteins in the kidney of the CTD and control groups, showing 79 downregulated DEPs and 92 upregulated DEPs (Figure 3B). Cluster analysis is shown in Figure 3C. GO enrichment analyses revealed that DEPs were associated with responses to the negative regulation of apoptosis, oxidative stress, glutathione metabolism, and lipid metabolic processes. Molecular function annotations included protein binding, enzyme binding, catalytic activity, D-glucose transmembrane transporter activity, and N-acetyltransferase activity (Figure 3D). Functional annotation identified 13 significantly enriched pathways, including metabolic pathways, cofactor biosynthesis, glutathione metabolism, tryptophan metabolism, pantothenate and coenzyme A biosynthesis, proximal tubule bicarbonate regeneration, and folate biosynthesis (Figure 3E).

PPI network analysis of the 171 DEPs was performed in STRING (confidence score ≥ 0.7) and visualized in Cytoscape 3.10.1. Topological parameters (BC, CC, DC) were calculated using CytoNCA, and nodes with values ≥ median were selected as key targets. This analysis identified hub proteins including Slc2a2, Gclc, Hgd, Cbx5 and Slc5a2 in CTD-induced kidney injury (Figure 3F).

### 3.4. Network Pharmacology Combined with Multi-Omics

**Figure 4:**
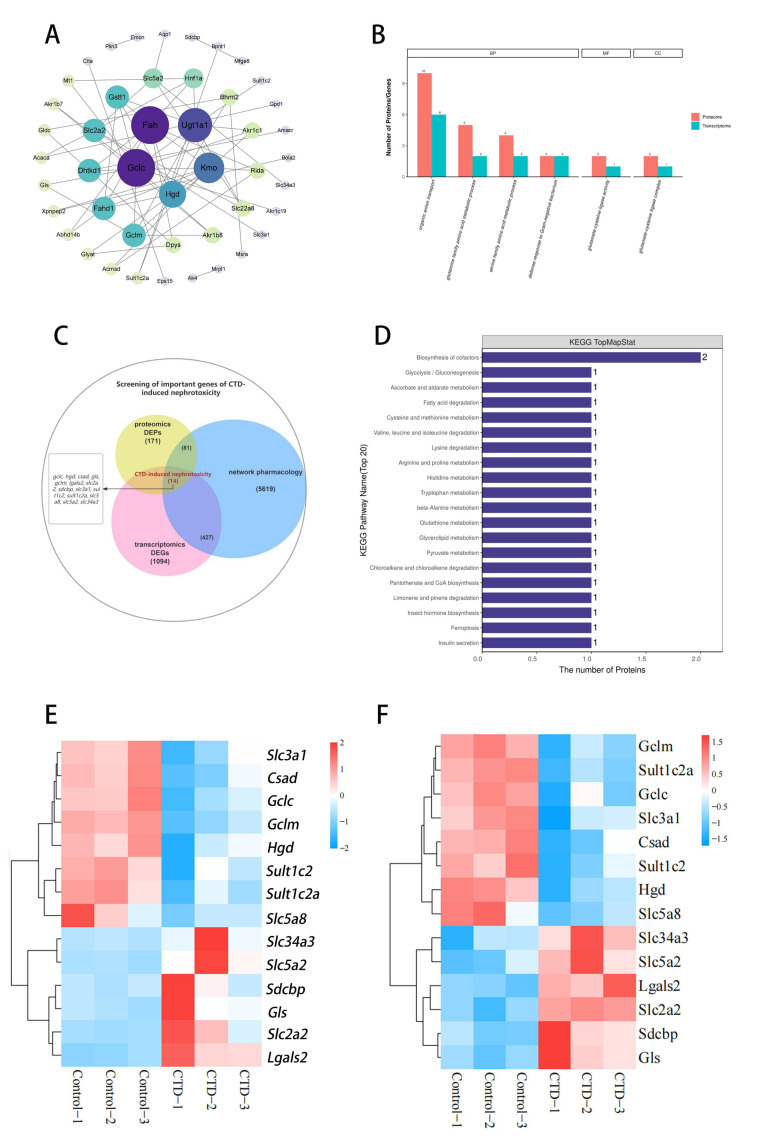
Analysis of network pharmacology combined with transcriptomics and proteomics. (**A**) Network pharmacology PPI analyses of CTD-induced kidney injury; (**B**) enriched associated GO entries (TOP5). Each column in the figure represents a GO secondary annotation entry, with red representing differentially expressed proteins and cyan representing differentially expressed genes. From left to right, the number of differentially expressed proteins is ranked from highest to lowest. The higher the column, the more active the GO secondary annotation item is in the test sample. (**C**) The Venn map was analyzed by proteomics, transcriptomics and network pharmacology. (**D**) The top 20 pathways were associated with the highest number of common targets; the common target of the two omics in the transcriptome (**E**) and proteome (**F**) clustering heat map, respectively. The blue block represents low relative expression value, and the red block represents high relative expression value.

Network pharmacology identified 5619 targets associated with CTD-induced kidney injury. Furthermore, a CTD-induced kidney injury target network was constructed, comprising 46 nodes and 65 edges (Figure 4A), indicating a multi-target mode of CTD nephrotoxicity. Topological analysis was performed to further explore core targets, revealing five core targets, including Fah, Gclc, Ugt1a1, Kmo, and Hgd. Further, we performed GO enrichment (Figure 4B) and KEGG pathway analysis (Figure 4D) on common DEPs/DEGs in transcriptomics and proteomics. The GO analysis of BPs revealed that these proteins/genes were mainly involved in organic anion transport, glutamate-cysteine ligase activity, glutamate-cysteine ligase complex, and amino acid metabolic processes of the glutamine family. KEGG pathway analysis revealed that these proteins/genes mainly involved the cofactor biosynthesis, glutathione metabolism, and cysteine metabolism. The KEGG enrichment pathway analysis identified the glutathione metabolism pathway as a common pathway.

Next, 5619 intersecting targets predicted by network pharmacology for CTD-induced kidney injury, with 171 DEPs and 1094 DEGs, yielded a total of 14 target genes (*gls*, *lgals2*, *slc2a2*, *sdcbp*, *slc34a3*, *slc5a2*, *slc3a1*, *hgd*, *sult1c2*, *gclc*, *gclm*, *slc5a8*, *sult1c2a* and *csad*) (Figure 4C). Cluster analysis was conducted on these 14 targets (Figure 4E,F). The results indicated that GLS, LGALS2, SLC2A2, and SDCBP were upregulated in both the proteome and transcriptome; SLC3A1, SULT1C2, HGD, GCLC, GCLM, SULT1C2A, CSAD, and SLC5A8 were downregulated in the proteome and transcriptome; SLC34A and SLC5A2 were downregulated in the proteome but upregulated in the transcriptome. This inconsistency may be attributed to post-transcriptional regulation, microRNA-mediated translational inhibition, altered protein stability, and differential protein degradation.

Among the 14 key targets associated with CTD-induced kidney injury, the top four core targets—HGD, GCLC, GCLM, and GLS—were screened out based on the ranking results of the sum of degrees in the transcriptome and proteome PPI analysis. These targets may be the key proteins causing CTD-induced kidney injury.

### 3.5. Molecular Docking and Western Blotting Verification

**Figure 5:**
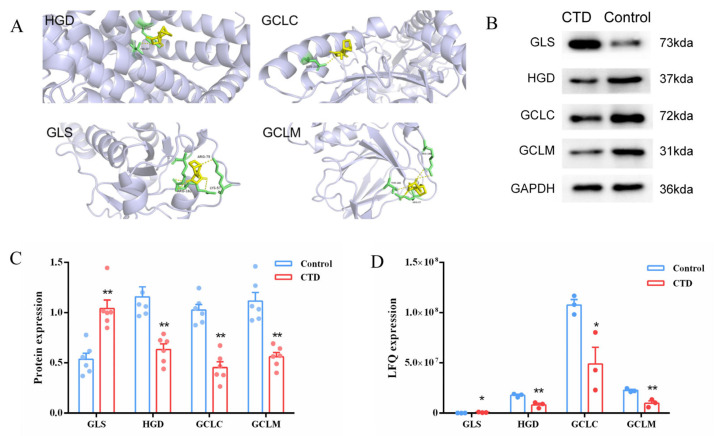
Molecular docking verification and relative protein expressions of overlapping proteins in rats with CTD-induced kidney injury. (**A**) Results of molecular docking between CTD and related nephrotoxic proteins; (**B**,**C**) the protein expression level of GLS, HGD, GCLC and GCLM by CTD (x ± SD, *n* = 6, ** represents *p* < 0.01). (**D**) Quantitative protein (LFQ) expression level of GLS, HGD, GCLC and GCLM by CTD ($\bar{x}$ ± SD, *n* = 3, * represents *p* < 0.05, ** represents *p* < 0.01).

Molecular docking was performed to evaluate the binding affinity of CTD to the four core targets identified via integrated omics analysis (Figure 5A). Binding energy values (Table 1) revealed favorable interactions (≤−5.0 kcal/mol) for HGD, GCLC, GLS and GCLM targets, indicating stable complex formation. Visualization analysis confirmed CTD binding to multiple active sites within each protein. Transcriptomic data indicated that compared with the control group, HGD, GCLC and GCLM protein levels were downregulated, and GLS mRNA levels were elevated exclusively in the CTD group (Figure 5D). Moreover, Western blots confirmed that CTD significantly downregulated protein expression of HGD, GCLC, and GCLM while upregulating GLS (Figure 5B,C), consistent with proteomic results.

## 4. Discussion

The development of kidney injury may be attributed to various factors, including nephrotoxic drugs, ischemia–reperfusion injury, and immune injury [24]. Underlying mechanisms include tubular epithelial cell injury, metabolic disturbance, mitochondrial dysfunction, excessive reactive oxygen species (ROS) production, cell cycle arrest, and dysregulated inflammatory responses [25,26,27]. CTD has been used to treat different types of tumors. However, clinical studies have shown that CTD can induce different degrees of kidney injury, with the detailed molecular mechanisms remaining unclear [28,29]. Therefore, this study explored the potential molecular mechanism of CTD-induced kidney injury ins rat based on transcriptomics, proteomics, and network pharmacology.

Scr and BUN are established biomarkers of renal function, with elevated levels reflecting tubular epithelial cell damage [14]. Biochemical analysis revealed that the levels of Scr and BUN were obviously elevated after the CTD treatment. On the other hand, glomerular and tubular congestion were the most notable pathological features of kidney injury [30]. Our results demonstrated that glomerular tuft retraction and the cloudy swelling of tubules were remarkably augmented in the CTD group. These findings suggest that CTD could cause toxic damage to the kidney.

Integrative analyses of transcriptomics, proteomics and network pharmacology enable systematic dissection of complex biological systems, which turn the mode of “one target and one pathway” into “multiple targets and multiple pathways” [31,32,33]. In this work, 1094 DEGs and 171 DEPs were identified, and 14 overlapping targets were screened following CTD intervention. The biological processes of CTD-induced kidney injury were predominantly related to organic anion transport, glutamate-cysteine ligase activity, the glutamate-cysteine ligase complex, and glutamine family amino acid metabolic processes. Meanwhile, we found that the kidney injury activity of CTD was involved in multiple pathways, including cofactor biosynthesis, glutathione metabolism, and cysteine metabolism.

Through transcriptomics, proteomics and network pharmacology analyses, we found three significant downregulation targets (HGD, GCLC and GCLM) and one upregulation target (GLS) in CTD-induced kidney injury. These four targets were further validated using Western blotting in protein levels, suggesting that they may play important roles in alleviating CTD-induced kidney injury.

GLS is a crucial enzyme that catalyzes the decomposition of glutamine into glutamate and ammonia, thereby regulating glutamine metabolism, oxidative stress, and acid-base balance [34,35,36]. Further research revealed that CTD affects glutamate metabolism disorder and induces kidney injury by upregulating GLS. HGD is a key enzyme in the metabolic pathways of phenylalanine and tyrosine. Its functional deficiency or abnormal activity can lead to abnormal accumulation of homogentisic acid (HGA) in the body, thereby leading to Alkaptonuria (AKU) [37]. HGD is involved in tyrosine catabolism, and its reduced expression may lead to the accumulation of toxic metabolites, which in turn promote oxidative stress and affect cellular redox balance [38]. In the HGD knockout mouse model, HGA deposition caused mitochondrial dysfunction in renal tubular epithelial cells and increased MDA and TNF-α [39]. In this study, CTD was found to downregulate the expression of HGD, which suggests that CTD may exacerbate oxidative damage by reducing the consumption of glutathione. Multi-omics enrichment analysis suggested that glutathione metabolism and oxidative stress pathways were significantly affected in CTD-induced kidney injury. However, although relevant tests were conducted in our previous studies, there is a lack of direct biochemical evidence in this study to support oxidative stress and impaired glutathione metabolism. Further experiments will be performed to detect these oxidative stress indexes to confirm this hypothesis.

Glutamate-cysteine ligase (GCL) is the rate-limiting enzyme for the synthesis of glutathione (GSH), and is composed of GCLC and GCLM [40]. The activity of GCL determines the efficiency of GSH biosynthesis, and plays a role in resisting oxidative stress, inflammation, and ferroptosis in the kidney [41]. In this study, the expression levels of GCLC and GCLM in the kidneys of rats in the CTD group decreased at the protein/gene level, suggesting that CTD may induce oxidative stress and cause kidney injury by down-regulating GCLC and GCLM, leading to glutathione metabolism disorder. Clinical studies have confirmed that enhancing the activity of GCL can effectively alleviate drug-kidney injury [42,43].

However, several limitations existing in this study should be overcome in future work. Firstly, in this study, we selected rats as the animal model to investigate CTD-induced kidney injury, which may pose species differences. Given the experimental constraints and limited sample availability, the detection of urinary markers was not performed in the current study. Future investigations will include the measurement of urinary KIM-1, NGAL, and proteinuria to enable a more comprehensive and precise evaluation of CTD-induced kidney injury. Secondly, we identified key proteins and verified their expression; however, these four targets have not yet undergone positive and negative verification such as pharmacological intervention. In the next step, it will be necessary to use RNA interference or agonists/inhibitors to clarify the signal transduction pathway through which CTD regulates kidney injury.

## 5. Conclusions

In summary, this study identified GCLC, GCLM, HGD and GLS as key targets in CTD-induced kidney injury using an integrated multi-omics strategy. CTD inhibits the glutathione metabolic pathway by downregulating GCLC and GCLM, leading to excessive oxidative stress and consequent renal tubular damage. These findings deepen our understanding of CTD-induced nephrotoxicity and provide novel targets for the development of preventive and therapeutic strategies.

## Figures and Tables

**Table 1:** Binding energy values of CTD-induced kidney injury.

Proteins	PDB ID	Binding Energy Values (kcal/mol)
HGD	2DDH	−6.5
GCLC	3A9Y	−5.6
GCLM	4IEV	−5.8
GLS	3HL4	−6.4

## Data Availability

The original contributions presented in this study are included in the article. Further inquiries can be directed to the corresponding authors.
